# Notes and Notices

**Published:** 1892-07

**Authors:** 


### NOTES AND NOTICES.

OBSTACLES TO HOMŒOPATHY.—Dr. Jousset, the celebrated French physician, in a speech delivered by him at a banquet given by the homœopathic doctors of Paris to celebrate the anniversary of Hahnemann, declared that the greatest drawback to the practice of this special branch of medicine in France was that it was not officially recognized, and that homœopathic doctors are looked upon in the light of outsiders and sectarians by the medical profession. He announced that in America there were at least twenty thousand homœopathic doctors, while in fair France there were only three hundred and fifty, and this in spite of the marvelous results during the cholera epidemic of 1848–49 of the Hahnemann method of treatment. Dr. Jousset complains that the medical hospitals are practically closed to them, and that the doctors of the allopathic school do all in their power to frighten patients from seeking relief in homœopathic treatment.—*The Illustrated American*, June 11th, 1892.

THE HOMŒOPATHIC MEDICAL SOCIETY OF WISCONSIN held its twenty-eighth annual session at Milwaukee, June 7th, 8th, and 9th, 1892. The officers are: A. G. Leland, M. D., of Whitewater, President; Joseph Lewis, Jr., M. D., First Vice-President; S. J. Martin, M. D., of Racine, Second Vice-President; A. R. F. Grob, M. D., Milwaukee, Recording Secretary; C. J. Steele, M. D., Milwaukee, Corresponding Secretary; E. W. Beebe, M. D., Milwaukee, Treasurer.

THE WOMEN'S HOMŒOPATHIC HOSPITAL.—On April 26th The Women's Homœopathic Association of Pennsylvania opened to the public the Lippe Memorial Pavilion. This is a special building for the accommodation of such cases as it is not advisable to place in the general hospital. The pavilion is eighty-four feet in length by thirty feet in width. It is built of the best quality stretcher brick, with double walls, there being a three and a half inch air space between. It contains one large ward and two private rooms for patients. The ward has four open fireplaces. The private rooms are also furnished with fireplaces. All windows are double with transoms over them in the ward and a lantern top to make the ventilation more perfect. This pavilion is heated and ventilated by the Sturtevant fan and heater. The entire air of the pavilion can be changed in a few minutes.

There are rooms for physicians and nurses attached to this building and for the help. Linen closets, bath-rooms, etc., all constructed according to the best sanitary rules. There are no angles in any part of the building. All doors and windows are of oak.

In the basement, which is light and airy, is a diet kitchen with all modern appliances. A laundry furnished with disinfecting tank, tubs, etc., where the clothing and bedding for patients will be laundried; also an ironing-room; a dining and a sitting-room for the doctor and nurses. In fact every thought has been given to make this pavilion perfect as possible and thoroughly sanitary in every way and comfortable for all the inmates.

Such a building seems a fitting memorial to Dr. Lippe, to whom it is dedicated. A tablet will be placed in the hall of the pavilion, bearing the following inscription: "In memory of Adolph Lippe, M. D., a consistent follower of Hahnemann. A man who had the courage of his convictions."

Northern Indiana and Southern Michigan Homœopathic Medical Association held its second semi-annual meeting Tuesday, May 10th, in South Bend, Ind., Dr. C. H. Myers in the chair. Members present were Drs. A. L. Fisher, Porter Turner, and H. A. Mumaw, Elkhart; John Borough, Mishawaka; I. O. Buchtel, Auburn; C. H. Myers, W. D. Chaffee, R. L. S ine, and Julia Godfrey, of South Bend. After roll call and prayer by Rev. A. B. Chaffee, the minutes of the previous meeting were read by the Secretary, Dr. H. A. Mumaw. Approved. Drs. W. A. Crandall, of Sturgis; J. C. Hunsinger, and S. N. Devor, Elkhart, and H. Hammond were elected. Dr. A. L. Fisher read a paper entitled "A Nameless Case and Relief from an Operation." Dr. John Borough, chairman of the Bureau of Obstetrics and Gynæcology, read a paper on the "Third Stage of Labor." Dr. W. D. Chaffee read a paper on "Membraneous Dysmenorrhoea." Dr. Julia Godfrey, chairman of the Bureau of Pædiatrics, read a paper on the "Care of Infants." Dr. J. O. Buchtel reported an interesting case of laryngismus stridulus, cured by Nux-vomica. Dr. R. N. Morris read a paper on the "Surgical Treatment of the Prepuce, and Cases Treated, with Results." A number of important papers were not read for want of time.

Election of officers for the ensuing year resulted as follows: President, Dr. A. L. Fisher; First Vice-President, Dr. I. O. Buchtel; Second Vice President, Dr. J. P. Siegfred, White Pigeon; Secretary, Dr. H. A. Mumaw; Treasurer, D. W. B. Kreider, Goshen. The next meeting will be held at Constantine, Mich., on the second Tuesday of September, 1892.

Dr. Olin M. Drake has removed from Ellsworth, Maine, to 70 Huntingdon Avenue, Boston, Mass.

Dr. Charles P. Beaman, from Ridgedale, Tenn., to 304 McCallie Avenue, Chattanooga, Tenn.

Dr. F. H. Lutze has removed from 133 Clymer Street to 232 Ross Street, Brooklyn, N. Y.

Dr. J. W. Thomson has removed from 248 W. Sixteenth Street to "The Irvington," 136 W. Sixteenth Street, New York.

Dr. A. Quackenbush has located at 401 Michigan Street, Buffalo, N. Y.

Dr. S. Mills Fowler has removed from Gainesville, Texas, to 3120 Wabash Avenue, Chicago.

Prof. A. L. Frothingham has located for the summer at Norfolk, Conn.

Removals.—Dr. F. F. Blanki, from Bœger's store, Mo., to 1600 Burd Ave., St. Louis, Mo.; Dr. R. O. Harris, to Carrollton, Mo.; Dr. Thomas W. Dike, from Newton Centre, to Dedham, Mass.; Dr. J. W. Cartlich, from Carrollton, to 1659 Madison Ave., Kansas City, Mo.; Dr. Mary Brower, has removed, for the summer months, from New York, to Walter's Park, Berks Co., Penna.; Dr. Frances M. Morris is located at 138 Marlboro' St., Boston, her former address was Princeton, Mass.

The Dios Chemical Co., of 914 Locust Street, St. Louis, Mo., issue a colored chart of Cerebral Localization, which they mail free to physicians on application.

Malted Milk.—Many physicians are recommending the use of Horlick's Malted Milk as a table drink in place of tea, coffee, cocoa, etc. The evil effects of the long continued use of tea or coffee are well known, but the difficulty has been to provide a pleasant and satisfactory substitute. Malted Milk served either hot or iced makes one of the most pleasant, refreshing, and nutritious drinks imaginable, little if any more expensive than the ordinary drinks, and of course far more healthy and nutritious. Does not stimulate but aids digestion. Prepared by simply adding water. Address the Malted Milk Co., Racine Wis., for samples.

The Yale Chair.—St. Louis, Mo., July 26th, 1892. I now am the owner of the Yale Chair, and I do not see how there could be anything more perfect than it is. I will be glad to recommend the Yale to any one that stands in need of a chair. H. L. Henderson, M. D.

The Weekly Bulletin.—The current issue of *The Weekly Bulletin of Newspaper and Periodical Literature*, published at 5 Somerset St., Boston, is twice its usual size, containing a classified index of 1,300 articles from recent numbers of the periodical press.

The *Bulletin* catalogues the important articles in the leading daily and weekly papers and the monthly magazines of the United States and Canada, including The Homœopathic Physician. Its value to readers, writers, and students is sufficiently indicated by its title, and, although still in its first volume, its success is evidenced by the current issue is a surprise to no one acquainted with its plan and purpose.

A Bureau of Service and Information for the exclusive use and benefit of visiting physicians and surgeons and their families will be opened in Chicago during the great Exposition of 1893 by Messrs. Chas. Traux Greene & Co., dealers in physicians' supplies, 75 and 77 Wabash Avenue, Chicago. Ample room will be provided for the successful operation of each department, and additional space set aside for the use of the secretaries and other officers of medical societies and conventions. No charge will be made for the services here offered, and all who are legitimately engaged in the practice of medicine and surgery will be made welcome. The services offered are as follows: Registration.—By registering name, college, and date of graduation, residence when at home, and hotel and boarding-house while in the city, telegrams and mail matter can be promptly forwarded and correct addresses furnished to all inquiring. Hotels and boarding-houses.—A list of leading hotels and boarding-houses will be kept, with location, description, and rates. Telegrams.—For these we will receipt if requested or assist (by means of our registry) in their speedy delivery. Postal Benefits.—A miniature post-office will be established, so that mail matter may be addressed in our care. Banking Facilities.—Cash will be paid out during banking hours from currency deposited with us and from funds forwarded us direct from banks. Money sent us by banks for credit should be accompanied by signature of depositor. Checks and drafts will not be cashed, and will be received only for collection. Telegraph, Telephone, Stenographic, District Messenger, Livery, Cab, Express, Baggage, and Freight Service arranged for in the building and legitimate rates secured. Check and Cloak Room.—Parcels and small packages will be received and checks issued for the same. Headquarters for Physicians.—A reading and reception room, with writing facilities and stationery will be provided, where physicians may meet their friends, attend to correspondence, etc. Purchasing Department.—Theatre, exposition, sleeping-car and railway tickets will be secured, and assistance rendered in purchasing goods in all lines of trade. Office Room and Desks, in or adjoining the general headquarters, will be provided for the secretaries and other officers of medical societies and conventions. Interpreters.—German, French, Spanish, and other interpreters will be permanently located in the building. These privileges will be granted to physicians and surgeons (and their families) only—college and date of graduation required on registration.

The Calcium Salts in Nutrition.—The Calcium Salts, both phosphate and carbonate, are indispensable in the formation and life of animals as of plants. They not only form the mineral part, and, as it were, framework of the bones and teeth, but are also integral parts of all the elements of the body, principally of the brain, marrow, nerves, muscles, and blood—in short, the support of the organic substance of all the cells. Indispensable to pregnant or nursing women, who, in addition to supporting their own bodies, are compelled to furnish material for the formation and development of a new organism. Likewise, of the greatest necessity for children during their period of growth.

Insufficiency of these principles must produce weakening of the constitution and dental caries on the part of both mother and infant—curvature of the bones, late and painful dentition, and a state of general debility.

It has been shown by numerous chemical analyses of the milk of nursing women that very often it is deficient in Calcium salts, notably phosphate—especially is this true of residents in the city. The cause of this, no doubt, is that during pregnancy the digestive disorders, which are so frequent, prevent an active assimilation of these principles in the state in which they are contained in the food.

The idea of adding some extra Calcium salts to the food suggests itself spontaneously. But the difficulty of making the compound enter into the economy is well known. When mixed with the food they ordinarily pass as foreign bodies into the salts of the urine, without benefiting the organism.

But as albuminated Calcium, both phosphate and carbonate, as exhibited in Proteinol—a food composed of the entire egg, digestible fat, brandy, and maltose is the form in which nature seems to have put these salts in small quantities in eggs, milk, grain, etc.—they are retained and utilized in the organism.

The use of Proteinol during pregnancy permits the birth of living and vigorous infants from mothers with a history of abortion, or whose children had hitherto come into the world weak, and had succumbed soon after birth.

Again, under the use of Proteinol pregnancy is more easily endured; the pregnant woman loses neither her strength nor her good general health and having arrived at term is in splendid condition for being an excellent nurse.

Moreover, the milk of women taking Proteinol is more abundant, is much richer in fat and the lime salts, seemingly so necessary to metabolism. Their children improve in health, their dentition is easy, and their growth rapid.—*Medical Advance.*

The Medical Visitor.—We desire to inform our readers that we will club The Homœopathic Physician with *The Medical Visitor* for the sum of *Three Dollars* net, cash with the order. *The Medical Visitor* is a pure Hahnemannian journal published in Chicago.

Homœopathy in Hungary.—The Government of Austria and Hungary, in consideration that the fourth part of the population are homœopathists, notwithstanding the doctors of the opposition established in 1870 an official denunciation of Homœopathy in the University of Buda-Pesth which is in charge of Professor Bakody, in the large hospital of the same city "St. Roque" two wards with sixty beds have been placed at the services of the homœopathists. This measure at first provoked the ire of the allopathic physicians and pharmacists, but it was soon calmed, and the two systems have continued thus for twenty-one years without sustaining any recrimination, to the great joy of the patients.—*Revista Hom.*, January, 1892.

The International Hahnemannian Association held its annual meeting at Narragansett Pier, June 21st, 22d, 23d, 24th. The session was most interesting and a large number of valuable papers was presented.

As the stenographer, Dr. J. B. S. King, one of the editors of *The Medical Advance*, has engaged to furnish us a copy of his notes, we shall ask our readers to be content with this short notice of the meeting as the full proceedings will be laid before them from time to time in installments the same as in previous years. The officers elected were as follows:

President, Dr. Edward Rushmore; Vice-President, Dr. T. S. Hoyne; Secretary, Dr. S. A. Kimball; Corresponding Secretary, Dr. Samuel Long; Treasurer, Dr. Frank Powel. Board of Censors, Dr. Wm. Wesselhœft, Dr. Edward Adams, Dr. H. C. Allen, Dr. F. S. Davis, Dr. A. R. Morgan.

SILICEA IN THE TREATMENT OF CANCER.—Some time ago a patient came to Dr. Formica who had a cancer in the breast. The patient was so diseased that there was no hope of any cure. Dr. Formica, though knowing this, yet took charge of the case, and having given several remedies that responded to the dominant symptoms, adopted as the principal remedy Silicea. The benefit was so great that he was able to give hopes of future recovery.

We have here a note from *The Homœopathic Recorder* that justifies this treatment: "Some years ago I treated a patient who had two scirrhus cancers, —very painful—in the left breast of a very unpleasant aspect. The progress of the disease made an operation indispensable, and at my indication Dr. Schuts was called, who considered an anæsthetic unnecessary, which was also my opinion, and in thirteen minutes the operation was accomplished."

We left together, and during our walk he told me the following: "About three years ago I was called to see Prince S., who had a cancer. The result was not satisfactory, although I did all I could, surgically, for two months. One day the Prince said to me that he wished to try Homœopathy, and suspend for a while my treatment. Dr. Fleishmann was then called. Weeks after, by chance I heard the Prince eulogize Homœopathy, and meeting Dr. Fleishmann on the street, asked him about the patient. My surprise was great when he told me that in three weeks after having charge of him he became convalescent, and the remedy he had employed was Silicea."

I strongly resolved to employ this remedy for the first case of the kind that would present itself. As my doctrine did not permit the use of infinitesimal doses, I triturated a grain of Silicea with one hundred of Sugar of Milk.

The first case that came to me was the wife of a high official, who had been operated upon for the second time some weeks before, and all the indications proved that the malignant character of the disease had not disappeared. After two weeks of my treatment, which consisted of a dose in morning and another in the evening, the aspect of the ulcer was very much improved; three weeks later it closed completely, and the patient was cured.

Since then I have always used Silicea for scirrhus, some have been operated on and others have not, and the result has always been satisfactory. I beg of you to give from this hour your patient this remedy prepared in the same strength by the apothecary of the patient I did thus: In six weeks from the use of Silicea that ulcer, so large, was entirely cured, and to-day after more than twenty years it has not re-appeared.—DOCTOR IRSCH.

We can, therefore, recommend the use of Silicea in cancerous ulcers as a hopeful medicine, now that its pathogenetics are aggregated with the clinical cases quoted above.

Translated from the *Revista Homœopetico*, Marzo y Abril, 1892.

